# Sortilin levels correlate with major cardiovascular events of diabetic patients with peripheral artery disease following revascularization: a prospective study

**DOI:** 10.1186/s12933-020-01123-3

**Published:** 2020-09-25

**Authors:** Federico Biscetti, Elisabetta Nardella, Maria Margherita Rando, Andrea Leonardo Cecchini, Nicola Bonadia, Piergiorgio Bruno, Flavia Angelini, Carmine Di Stasi, Andrea Contegiacomo, Angelo Santoliquido, Dario Pitocco, Raffaele Landolfi, Andrea Flex

**Affiliations:** 1 Department of Internal Medicine, Fondazione Policlinico Universitario A. Gemelli IRCCS, Largo Agostino Gemelli 8, Roma, 00168 Italia; 2 Internal Medicine and Vascular Diseases Unit, Roma, Italia; 3 Laboratory of Vascular Biology and Genetics, Department of Translational Medicine and Surgery, Roma, Italia; 4 Emergency Medicine, Roma, Italia; 5 Cardiac Surgery Unit, Roma, Italia; 6 Department of Radiology, Roma, Italia; 7 Università Cattolica del Sacro Cuore, Roma, Italia; 8 Angiology Unit, Roma, Italia; 9 Diabetology Unit, Roma, Italia

**Keywords:** Diabetes mellitus, Peripheral artery disease (PAD), Sortilin

## Abstract

**Background:**

Peripheral artery disease (PAD) represents one of the most relevant vascular complications of type 2 diabetes mellitus (T2DM). Moreover, T2DM patients suffering from PAD have an increased risk of major adverse cardiovascular events (MACE) and major adverse limb events (MALE). Sortilin, a protein involved in apolipoproteins trafficking, is associated with lower limb PAD in T2DM patients.

**Objective:**

To evaluate the relationship between baseline serum levels of sortilin, MACE and MALE occurrence after revascularization of T2DM patients with PAD and chronic limb-threatening ischemia (CLTI).

**Research design and methods:**

We performed a prospective non-randomized study including 230 statin-free T2DM patients with PAD and CLTI. Sortilin levels were measured before the endovascular intervention and incident outcomes were assessed during a 12 month follow-up.

**Results:**

Sortilin levels were significantly increased in individuals with more aggressive PAD (2.25 ± 0.51 ng/mL vs 1.44 ± 0.47 ng/mL, *p* < 0.001). During follow-up, 83 MACE and 116 MALE occurred. In patients, who then developed MACE and MALE, sortilin was higher. In particular, 2.46 ± 0.53 ng/mL vs 1.55 ± 0.42 ng/mL, p < 0.001 for MACE and 2.10 ± 0.54 ng/mL vs 1.65 ± 0.65 ng/mL, *p* < 0.001 for MALE. After adjusting for traditional atherosclerosis risk factors, the association between sortilin and vascular outcomes remained significant in a multivariate analysis. In our receiver operating characteristics (ROC) curve analysis using sortilin levels the prediction of MACE incidence improved (area under the curve [AUC] = 0.94) and MALE (AUC = 0.72).

**Conclusions:**

This study demonstrates that sortilin correlates with incidence of MACE and MALE after endovascular revascularization in a diabetic population with PAD and CLTI.

## Background

Cardiovascular complications of type 2 diabetes mellitus (T2DM) represent a social and economic burden quantifiable in almost 40 billion dollars considering the United States only [1]. This is due to the fact that diabetic patients may experience major adverse cardiovascular events (MACE) and major adverse limb events (MALE), which worsen quality of life and work capacity, leading often to death [2]. Among vascular complications of diabetes, peripheral artery disease (PAD) is one of the most significant and invalidating [3]. In fact, patients with PAD have a substantial decline in terms of life quality and expectancy [4]. Length and quality of life in PAD patients are not only related to lower limb events, but also to subsequent MACE, since PAD is a risk factor for myocardial infarction, stroke and cardiovascular death [2]. The most updated guidelines recommend a multidisciplinary approach, which aims to reduce modifiable risk factors and to promptly treat complications [1, 5]. Pharmacological therapy includes hypoglycemic agents, hypertension drugs, antiplatelet agents and lipid-lowering drugs [5]. Despite the best therapeutic approach, many patients experience chronic limb threatening ischemia (CLTI) [6]. In this circumstance, revascularization is indicated [6]. Whenever possible, endovascular lower limb revascularization (LER) ensures satisfactory results with a good risk/benefit ratio [6]. However, during the period following LER, an important percentage of patients experience MACE and MALE [2, 7]. Despite the best risk stratification at time of LER, it is impossible to predict time and occurrence of vascular complications [6]. In this scenario, biomarkers for risk stratification, offering personalized follow-up strategies, are essential [8].

Among possible biomarkers, recently identified and widely studied, is sortilin, a protein encoded by the SORT1 gene, located on chromosome 1 [9]. This protein was subsequently found in the hepatocytes, where it plays a fundamental role in low-density lipoprotein cholesterol (LDL-C) trafficking [10]. In fact, sortilin promotes the LDL-C uptake into hepatocytes, through a mechanism independent of the LDL receptor [11, 12]. Furthermore, sortilin is involved in the entry pathway of LDL into macrophages, contributing to foam cell formation [13], platelet activation [14] insulin resistance and diabetes [15–17]. Starting from this evidence, several studies investigated the relationship between sortilin and vascular complications of T2DM [14, 18, 19]. In particular, circulating levels of this protein are increased in diabetic patients with coronary artery disease (CAD) [20]. More recently, we observed that sortilin levels correlate with the presence and severity of PAD in a cohort of diabetic patients [21].

Given the available reports, we postulate that sortilin levels may impact the incidence of cardiovascular complications after LER.

The aim of this study is to evaluate the relationship between baseline serum levels of sortilin and vascular outcomes, in particular MACE and MALE, after LER intervention in T2DM patients with PAD and CLTI.

## Research design and methods

### Study design

We performed a prospective non-randomized study, approved by the Ethics Committee of the Fondazione Policlinico Universitario A. Gemelli IRCCS. The aim of the study was to assess the relationship between sortilin levels at time of LER and the incidence of MACE and MALE, in a cohort of T2DM patients with PAD and CLTI. All included patients gave consent to participate in the study, which adhered to the principles of the Declaration of Helsinki.

### Study population and clinical assessment

The study population included 230 patients with T2DM complicated by PAD and CLTI, which required revascularization at the Fondazione Policlinico Universitario A. Gemelli IRCCS, Roma, Italy. The patients were consecutively enrolled during the period between 20/05/2018 and 30/03/2019. The inclusion criteria included: age of at least 18 years, T2DM diagnosis present for at least 1 year, an Ankle/Brachial Index (ABI) of less than 80, at least one lower limb stenosis greater than 50% documented by Ultrasound Color Doppler (US), stage 4 or 5 PAD diagnosis according to the Rutherford classification, presence of CLTI as previously described [7], and indication for LER of the target arterial stenosis. The exclusion criteria were: statin therapy within the previous 3 months, revascularization of the lower limb in the previous 3 months, diabetic foot ulcers with signs of active infection or osteomyelitis, diabetic peripheral neuropathy, homozygous familial hypercholesterolemia, absolute contraindication to antiplatelet therapy, thrombophilia, active cancer, active autoimmune disease, liver disease at functional status B or C according to Child–Pugh classification, life expectancy lower than 12 months, pregnancy.

The wound, ischemia, foot infection (WIfI) classification system was used to classify and stratify patients with diabetic foot ulcer. When necessary, radiological examination was performed to rule out osteomyelitis. Diabetic peripheral neuropathy has been excluded as previously described [7]. PAD was defined according to the criteria of the Society for Vascular Surgery and the International Society for Cardiovascular Surgery [22]. All patients underwent lower limb US. For patients with ABI of 1.40 or higher, US has been also used to confirm significant stenosis in cases of arterial calcification.

The following clinical and laboratory data were collected for all patients. Complete clinical history, in particular, history of CAD, history of cerebrovascular disease (CVD), hypertension, smoking status, Body Mass Index (BMI), blood tests as described below.

At time of LER, all patients were on single antiplatelet therapy and, after revascularization, took double antiplatelet therapy (DAPT) for 1 month.

After LER, patients started lipid-lowering therapy to reach an LDL-C target lower than 70 mg/dl.

### Endovascular revascularization procedure and follow-up

LER was performed as previously described [7]. Twenty-two (8.73%) out of 252 patients experienced primary treatment failure after revascularization and were excluded from follow-up. Angioplasty and, if indicated, arterial stenting were defined as successful if the residual arterial stenosis was less than 30% of the lumen [23]. No major peri-operative complications were registered, according to the definitions of the Society of Interventional Radiology [23]. During the 12 month follow-up, patients were evaluated at 1, 3, 6 and 12 months after LER to assess the incidence of MACE and MALE outcomes. MACE was defined as composite of myocardial infarction, stroke and cardiovascular death. MALE referred to a composite outcome of acute limb ischemia, major vascular amputations, limb-threatening ischemia leading to urgent revascularization [7].

### Blood test and biochemical analysis

On the day of LER, a blood sample was taken from all patients after a night of fasting. Glucose, creatinine, total cholesterol, LDL-C, triglycerides and glycated hemoglobin were evaluated. Renal function was calculated by modification of diet in renal disease (MDRD) formula to determine estimated glomerular filtration rate (eGFR). Serum was separated by centrifugation of blood samples and was stored at − 80 °C before every evaluation. A commercially available ELISA kit (RAB1709 SIGMA, Sigma-Aldrich) determined sortilin levels according to the manufacturer protocol. The intra and inter-assay coefficients of variation were 3.5 and 10.5%, respectively. The sensitivity, defined as the mean ± 3 SD of the 0 standard, was calculated to be 0.15 pmol/mL. For each patient, the serum levels were measured twice and the results were averaged.

### Statistical analysis

Demographic and clinical data were summarized as means (standard deviations) for continuous variables and counts (percentages) for categorical variables. Chi square and t-tests were used to compare groups, when appropriate. A log transformation was applied to the not normally distributed variables prior to performing other analyses. Sortilin levels were compared, when appropriate, with a Mann–Whitney, Kruskal–Wallis and Dunn’s Multiple Comparison. A multivariate stepwise logistic regression analysis, adjusted for traditional atherosclerotic risk factors and sortilin levels, was performed. The area under the receiver operating characteristics (ROC) curve was computed to test the predictive discrimination of MACE or MALE. The freedom from MACE according to the tertiles of sortilin levels was assessed using the Kaplan–Meier method and compared using the Log-Rank test. All analyses were performed using STATA version 14.0 for MacOS (Statistics/Data Analysis, Stata Corporation, College Station, TX, USA) and SPSS version 25.0 for MacOS (IBM Corporation, Armonk, NY, USA). Statistical significance was established at *p < *0.05.

## Results

### Characteristics of the study population

**Fig. 1:**
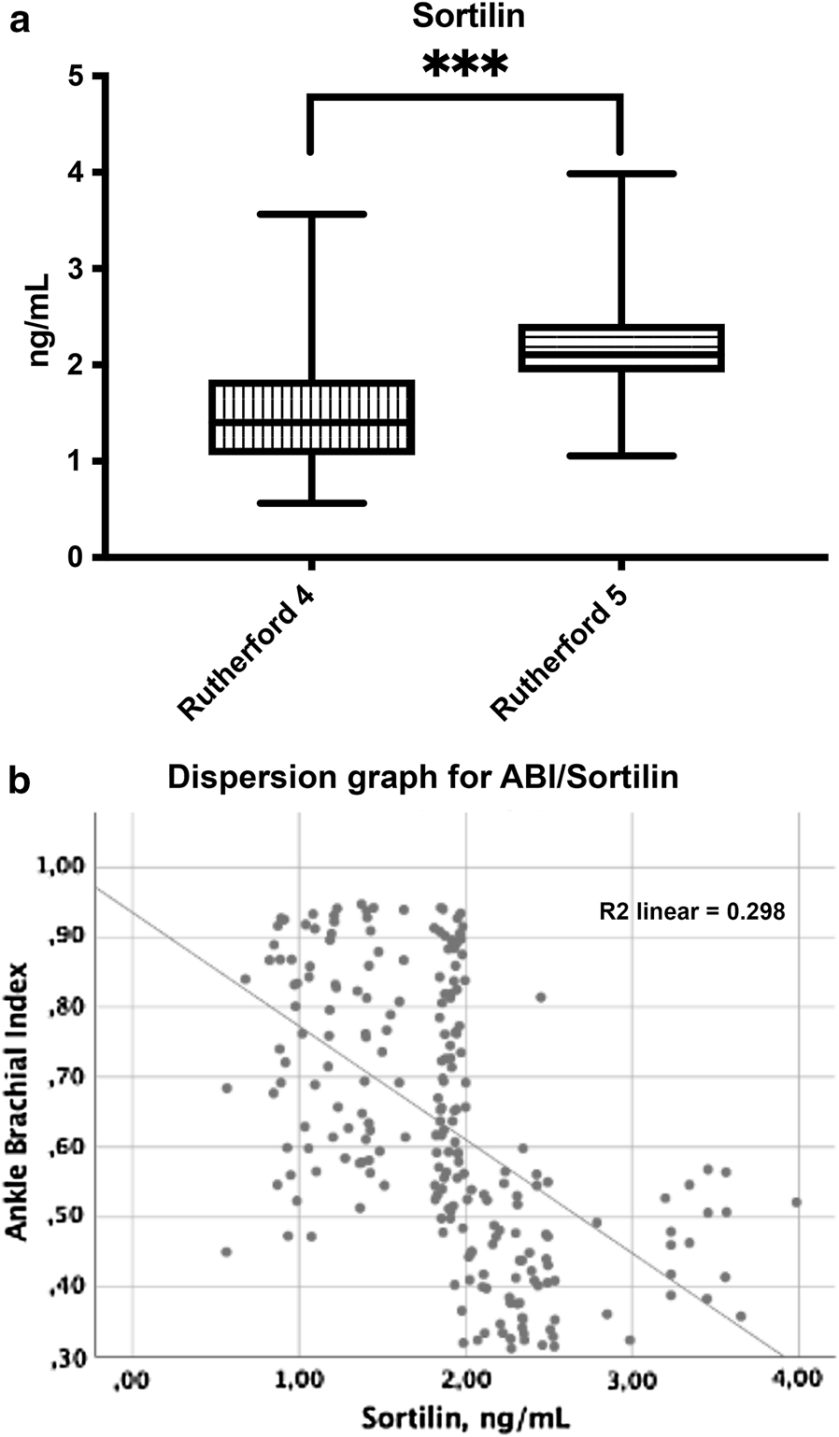
**a** Sortilin levels according to PAD severity. On the box plots, central lines represent the median, the length of the box represents the interquartile range and the lines extend to minimum and maximum values. ***P < 0.001. **b** Dispersion graph showing the correlation between Ankle Brachial Index (ABI) and serum levels of sortilin

We analyzed data of 230 patients in this study of which 142 (61.7%) were male. The mean age (SD) of the population was 71.1 (9.2) years with an average T2DM duration of 10.4 (2.5). We registered 77 active smokers (33.5%), 90 ex-smokers (39.1%) and the remaining 63 individuals (27.4%) had never smoked. The mean levels of glycated hemoglobin were 8.9% (2.0) and of LDL-C were 113.2 mg/dL (27.3). Considering PAD severity, 106 (46.1%) patients had Rutherford 4 disease and 124 (53.9%) patients had Rutherford 5 disease. The complete characteristics of the population are shown in Table 1. Mean sortilin levels were 1.9 ng/mL (0.6) and patients with severe PAD had higher levels than those with less severe PAD (2.25 ± 0.52 ng/mL vs 1.44 ± 0.48 ng/mL, *p* < 0.001) (Fig. 1a). Interestingly, we observed a significant inverse correlation between sortilin levels and the population's ABI values (Fig. 1b).

**Table 1:** Demographic characteristics of the study cohort at baseline

Number of patients	230
Age, years (SD)	71.1 (9.2)
Men, n (%)	142 (61.7)
Women, n (%)	88 (38.3)
Diabetes duration, years (SD)	10.4 (2.5)
BMI, kg/m^2^ (SD)	26.4 (1.8)
Oral antidiabetic agents, n (%)	130 (56.5)
Insulin, n (%)	171 (74.3)
High blood pressure, n (%)	165 (71.7)
Hypercholesterolemia, n (%)	149 (149)
Smoking status	
Never smoked, n (%)	63 (27.4)
Past smoker, n (%)	90 (39.1)
Current smoker, n (%)	77 (33.5)
ABI (SD)	0.63 (0.2)
Rutherford staging	
Stage 4, n (%)	106 (46.1)
Stage 5, n (%)	124 (53.9)
WIfI classification	
WIfI 010, n (%)	45 (19.6)
WIfI 020, n (%)	53 (23.0)
WIfI 110, n (%)	64 (27.8)
WIfI 120, n (%)	68 (29.6)
Previous coronary artery disease, n (%)	105 (45.7)
Previous cerebrovascular disease, n (%)	124 (53.9)
Total cholesterol, mg/dL (SD)	191.8 (28.2)
LDL cholesterol, mg/dL (SD)	113.2 (20.6)
Triglycerides, mg/dL (SD)	181.1 (27.3)
Glucose, mg/dL (SD)	120.7 (12.9)
HbA1C, % (SD)	8.9 (2.0)
eGFR, mL/min/1.73 m^2^ (SD)	71.1 (12.8)
Sortilin, ng/mL (SD)	1.9 (0.6)

Data are reported as means (standard deviation) for continuous variables and numbers (percentages) for categorical variables. *BMI* Body Mass Index, *ABI* Ankle Brachial Index, *WIfI* Wound, ischemia, foot infection, *LDL* low-density lipoprotein, *eGFR* estimated glomerular filtration rate

### Serum levels of sortilin and incidence of MACE at 12 months

All patients were followed up for a total of 12 months. During the follow-up we observed 83 MACE.

**Fig. 2:**
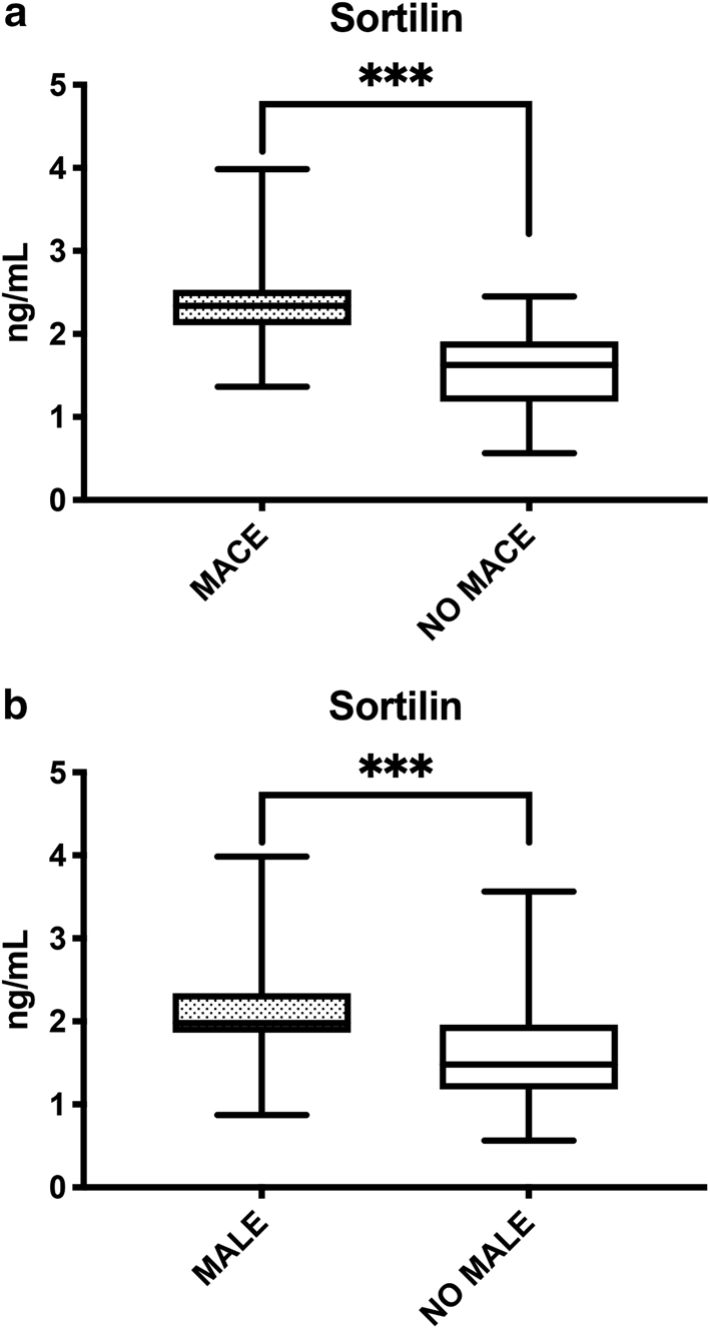
**a** Sortilin levels according to MACE. On the box plots, central lines represent the median, the length of the box represents the interquartile range and the lines extend to minimum and maximum values. ***P < 0.001. **b** Sortilin levels according to MALE. On the box plots, central lines represent the median, the length of the box represents the interquartile range and the lines extend to minimum and maximum values. ***P < 0.001

Patients experiencing MACE were mostly male (64, *p* < 0.001), suffering from hypertension (71, *p* < 0.001), hypercholesterolemia (65, *p* = 0.001) and were active (37, *p* = 0.007) or ex-smokers (44, *p* = 0.001). In addition, they had lower ABI (*p* < 0.001), higher total cholesterol (*p* < 0.001), LDL-C (*p* < 0.001) and sortilin levels (Fig. 2a) (*p* < 0.001), compared to patients without MACE. The characteristics of patients with or without MACE are fully reported in Table 2 and in Additional file 1. Figure S1.

**Table 2:** Demographic and clinical data of diabetic subjects with and without MACE

	NO MACE (n = 147)	MACE (n = 83)	*p* value
Age, years	71.17	71.11	0.961
Men:women, n	78.69	64:19	0
Body Mass Index, kg/m^2^	26.39	26.41	0.923
Diabetes duration, years	10.49	10.17	0.363
Ankle Brachial Index	0.72	0.47	0
High blood pressure, n (%)	94 (63.9)	71 (85.5)	0
Hypercholesterolemia, n (%)	84 (57.1)	65 (78.3)	0.001
CAD, n (%)	70 (47.6)	35 (42.2)	0.425
CVD, n (%)	80 (54.4)	44 (53.0)	0.837
Current smokers, n (%)	40 (27.2)	37 (44.6)	0.007
Past smokers, n (%)	46 (31.3)	44 (53.0)	0.001
Never smoked, n (%)	61 (41.5)	2 (2.4)	0
Rutherford 4, n (%)	96 (65.3)	10 (12.0)	0
Rutherford 5, n (%)	51 (34.7)	73 (88.0)	0
Total cholesterol, mg/dL	182.92	207.52	0
LDL cholesterol, mg/dL	103.55	130.33	0
Triglycerides, mg/dL	181.48	180.51	0.797
Glucose, mg/dL	120.12	121.59	0.405
HbA1C, %	8.93	8.71	0.423
eGFR mL/min/1.73 m^2^	71.36	70.60	0.669
Sortilin ng/mL	1.55	2.45	0

*BMI* Body Mass Index, *CAD* Coronary Artery Disease, *CVD* Cerebrovascular Disease, *LDL* low-density lipoprotein, *eGFR* estimated glomerular filtration rate. Statistical test performed with Student’s *t*-test or with Chi square test, when appropriate

**Fig. 3:**
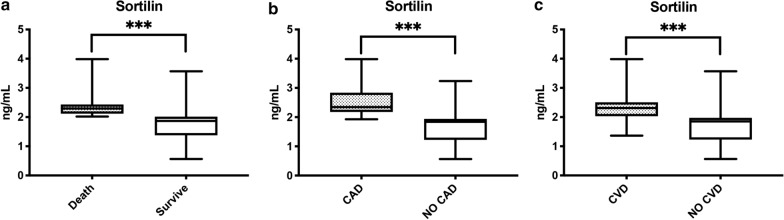
**a** Sortilin levels according to mortality. On the box plots, central lines represent the median, the length of the box represents the interquartile range and the lines extend to minimum and maximum values. ***P < 0.001. **b** Sortilin levels according to coronary artery disease (CAD) outcome. On the box plots, central lines represent the median, the length of the box represents the interquartile range and the lines extend to minimum and maximum values. ***P < 0.001. **c** Sortilin levels according to cerebrovascular disease (CVD) outcome. On the box plots, central lines represent the median, the length of the box represents the interquartile range and the lines extend to minimum and maximum values. ***P < 0.001

Considering the three aspects of MACE composite outcome, we noted a significant difference in terms of sortilin levels in each single event analyzed (death, CAD and CVD). In particular, the deceased patients had sortilin levels of 2.48 ± 0.56 ng/mL and the surviving patients 1.80 ± 0.61 ng/mL (*p* < 0.001) (Fig. 3a). Patients with CAD had sortilin levels of 2.56 ± 0.53 ng/mL and those without 1.63 ± 0.48 ng / mL (*p* < 0.001) (Fig. 3b). Patients with CVD had sortilin levels of 2.41 ± 0.56 ng/mL and patients without CVD 1.72 ± 0.57 ng / mL (*p* < 0.001) (Fig. 3c).

**Fig. 4:**
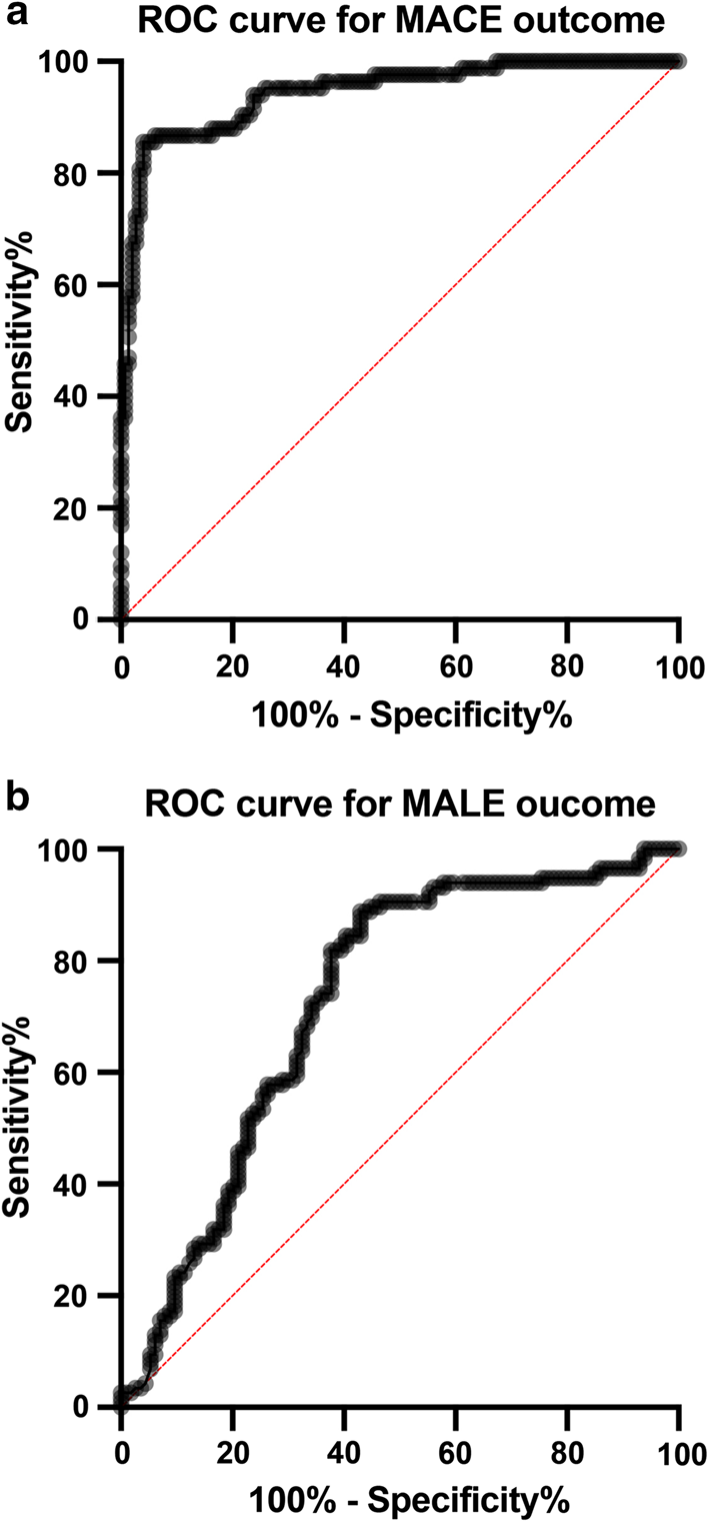
**a** ROC curve analysis to predict absence of MACE related to sortilin levels in T2DM showing an area under the ROC curve of 0.945 (*P* < 0.001). **b** ROC curve analysis to predict absence of MALE related to sortilin levels in T2DM showing an area under the ROC curve of 0.725 (*P* < 0.001)

The ROC curve, to predict the incidence of MACE based on sortilin levels, was elaborated and the area under the curve (AUC) was 0.94 (95% CI 0.91, 0.98, *p* < 0.001) (Fig. 4a).

**Fig. 5:**
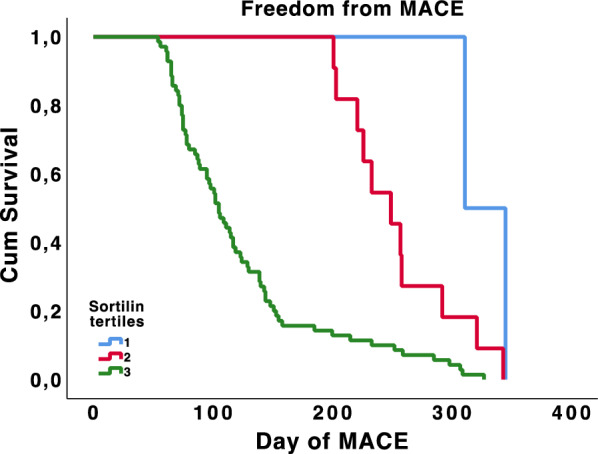
The freedom from MACE according to the tertiles of serum sortilin was estimated using the Kaplan–Meier method and compared using the Log-Rank test (*P* < 0.001). The tertiles of sortilin are listed in color code lines. Blue represents the first tertile (0.56–1.63 ng/mL; 2 events), red the second (1.64–2.00 ng/mL; 11 events), green the third (2.01–3.99 ng/mL; 70 events)

Sortilin levels were then divided into tertiles to compute the Kaplan–Meier curve shown in Fig. 5. We observed a significant difference between sortilin tertiles in terms of survival from MACE, and patients with the highest tertiles had a higher incidence of MACE compared to patients with lower tertiles (Log-Rank *p* < 0.001).

Multivariate analyses, after adjustment for traditional risk factors for cardiovascular events, showed that male sex (*p* = 0.05, 95% CI 0, 0.181), LDL-C values (p < 0.001, 95% CI 0.005, 0.011) and the serum levels of sortilin (*p* < 0.001, 95% CI 0.271, 0.457) were independent determinants of MACE in patients with CLTI subjected to LER (Table 3).

**Table 3:** Multivariable logistic regression for MACE

	Coef	St.Err	*t* value	*p* value	95% conf	Interval	Sig
Age	− 20.002	0.002	− 0.73	0.468	− 0.006	0.003	
Male sex	0.091	0.046	1.97	0.05	0	0.181	**
High blood pressure	0.036	0.05	0.72	0.474	− 0.063	0.135	
Hypercholesterolemia	0.014	0.047	0.31	0.76	− 0.078	0.107	
CAD	− 0.073	0.044	− 1.65	0.101	− 0.16	0.014	
CVD	0.016	0.044	0.35	0.724	− 0.071	0.102	
Current smokers	0.008	0.065	0.13	0.899	− 0.12	0.136	
Past smokers	− 0.053	0.066	− 0.81	0.418	− 0.183	0.076	
Never smoked	0						
LDL-C	0.008	0.001	6.07	0	0.005	0.011	***
FPG	0.003	0.002	1.61	0.11	− 0.001	0.006	
HbA1C	− 0.007	0.011	− 0.59	0.558	− 0.029	0.016	
Sortilin	0.364	0.047	7.72	0	0.271	0.457	***
Constant	− 1.422	0.314	− 4.52	0	− 2.041	− 0.802	***
Mean dependent var	0.361		SD dependent var			0.481	
R-squared	0.576		Number of obs			230.000	
F-test	24.592		Prob > F			0.000	
Akaike crit. (AIC)	143.845		Bayesian crit. (BIC)			188.540	

***p < 0.01, **p <0 .05

Multivariate analysis with similar adjustments was also performed to evaluate the incidence of distinct determinants of MACE, death, CAD and CVD. Notably, sortilin levels were also found to be independent determinants of death (*p* = 0.0.21, 95% CI 0.014, 0.169) (Additional file 1. Table S1), CAD (*p* < 0.001, 95% CI 0.236, 0.423) (Additional file 1. Table S2) and CVD (p < 0.001, 95% CI 0.126, − 0.336) (Additional file 1. Table S3).

### Serum levels of sortilin and incidence of MALE at 12 months

During the 12 months following the intervention of LER, 116 MALE occurred. Patients with MALE were more affected by blood hypertension (91, *p* = 0.023) and hypercholesterolemia (85, *p* = 0.007), were more ex-smokers (64, *p *< 0.001) and had a significantly reduced ABI compared to those without MALE (p < 0.001), who had higher LDL-C levels (p = 0.001) and higher sortilin levels (*p* < 0.001). The characteristics of patients with or without MALE are fully reported in Table 4 and in Additional file 1. Figure S1.

**Table 4:** Demographic and clinical data of diabetic subjects with and without MALE

	No MALE (n = 114)	MALE (n = 116)	*p* value
Age, years	71.20	71.09	0.93
Men:women, n	65:43	77:20	0.144
Body Mass Index, kg/m2	26.42	26.39	0.9
Diabetes duration, years	10.37	10.37	0.997
Ankle Brachial Index	0.68	0.59	0
High blood pressure, n (%)	74 (64.9)	91 (78.4)	0.023
Hypercholesterolemia, n (%)	64 (56.1)	85 (73.3)	0.007
CAD, n (%)	56 (49.1)	49 (42.2)	0.295
CVD, n (%)	66 (57.9)	58 (50.0)	0.230
Current smokers, n (%)	41 (36.0)	36 (31.0)	0.428
Past smokers, n (%)	26 (22.8)	64 (55.2)	0
Never smoked, n (%)	47 (41.2)	16 (13.8)	0
Rutherford 4, n (%)	78 (68.4)	28 (24.1)	0
Rutherford 5, n (%)	36 (31.6)	88 (75.9)	0
Total cholesterol, mg/dL	189.38	194.17	0.198
LDL cholesterol, mg/dL	108.37	117.99	0.001
Triglycerides, mg/dL	180.22	182.02	0.619
Glucose, mg/dL	120.31	120.99	0.689
HbA1C, %	8.72	8.98	0.324
eGFR mL/min/1.73 m^2^	71.69	70.49	0.479
Sortilin ng/mL	1.65	2.10	0

*BMI* Body Mass Index, *CAD* Coronary Artery Disease, *CVD* Cerebrovascular Disease, *LDL* low-density lipoprotein, *eGFR* estimated glomerular filtration rate. Statistical test performed with Student’s *t*-test or with Chi square test, when appropriate

The ROC curve was also elaborated to predict the incidence of MALE in relation to the basal of sortilin levels resulting in an AUC of 0.72 (95% CI 0.66, 0.80, *p* < 0.001) (Fig. 4b).

The multivariate analysis, after adjustment for the main cardiovascular risk factors, is shown in Table 5. The independent determinants of MALE after LER performed in T2DM patients with PAD and CLTI remained after adjusting for smoking habits (*p* = 0.003, 95% CI 0.099, 0.469) and sortilin levels (*p *= 0.037, 95% CI 0.009, 0.274).

**Table 5:** Multivariable logistic regression for MALE

	Coef	St.Err	*t* value	*p* value	95% conf	Interval	Sig
Age	− 0.002	0.003	− 0.60	0.547	− 0.009	0.005	
Male sex	0.012	0.065	0.18	0.854	− 0.117	0.141	
High blood pressure	0.08	0.071	1.12	0.265	− 0.061	0.22	
Hypercholesterolemia	0.104	0.067	1.55	0.124	− 0.029	0.236	
CAD	− 0.062	0.063	− 0.98	0.328	− 0.185	0.062	
CVD	− 0.083	0.062	− 1.33	0.186	− 0.206	0.04	
Current smokers	0.076	0.093	0.82	0.412	− 0.106	0.258	
Past smokers	0.284	0.094	3.03	0.003	0.099	0.469	***
Never smoked	0						
LDL-C	0.001	0.002	0.36	0.717	− 0.003	0.004	
FPG	0.001	0.002	0.33	0.745	− 0.004	0.006	
HbA1C	0.025	0.016	1.54	0.125	− 0.007	0.056	
Sortilin	0.141	0.067	2.10	0.037	0.009	0.274	**
Constant	− 0.2	0.448	− 0.45	0.655	− 1.083	0.682	
Mean dependent var	0.504		SD dependent var			0.501	
R-squared	0.206		Number of obs			230.000	
F-test	4.690		Prob > F			0.000	
Akaike crit. (AIC)	306.806		Bayesian crit. (BIC)			351.501	

***p <0 .01, **p <0 .05

## Discussion

The first result of our study is that sortilin levels correlate statistically significant with ABI and are higher in patients with lower ABI. Unsurprisingly, patients with a more severe PAD, at time of enrollment, had higher sortilin levels than those with less severe disease.

The management of cardiovascular complications of T2DM is an unresolved problem. In particular, MACE and MALE in T2DM patients with PAD and CLTI are very frequent [2] and are caused by pathological mechanisms, which can be relevant from a diagnostic point of view or considered an important therapeutic objective [8]. For example, cholesterol, in particular LDL-C, constitutes both a risk factor for atherosclerotic pathologies and a risk stratification biomarker, but it is also an important objective of medical therapy [24].

Among the molecules involved in LDL-C pathways, sortilin is a promising biomarker. In fact, sortilin is involved in LDL-C trafficking. On the one hand, a direct relationship between intrahepatic sortilin concentration and plasma LDL-C levels exists, both through an ApoB100-mediated mechanism [25] and increased secretion of proprotein convertase subtilisin/kexin type 9 (PCSK9) [26]. On the other hand, serum levels of sortilin may have a direct role in the atherosclerotic plaque formation, with an independent LDL-C mechanism [13]. In fact, the action of sortilin in immune cells, including macrophages, can worsen the development of atherosclerosis, with mechanisms not yet fully described, and in any case not exclusively dependent on the uptake of LDL-C [13, 19].

The role of sortilin in LDL metabolism is still unclear. Hepatic sortilin increases the clearance of LDL-C and reduces the secretion of very-LDL (VLDL) [27]; in contrast, studies performed in transgenic mouse models have shown the opposite effect [25, 28]. Therefore, specifically the role of circulating sortilin remains unclear or more precisely, if sortilin has a direct role on lipid metabolism and on the formation of atherosclerotic plaque, or if it represents an epiphenomenon useful as biomarker of cardiovascular risks in selected patients. In fact, substantial difference between data obtained on diabetic and non-diabetic populations exists. Notably, in a relatively small cohort of patients, sortilin levels were reduced in the diabetic compared to non-diabetic patients. Furthermore, the reduced levels were also associated with a worse metabolic profile. However, it should be noted that the potentially confounding and influencing effects of some drugs, such as metformin, have not been studied in this report [29].

Finally, a further element worthy of analysis is centered on the substantial difference existing between the two pathological conditions, CAD and PAD. In fact, an important distinction has been described in terms of comorbidity between T2DM patients with CAD or CLTI requiring revascularization [30]. This discrepancy could contribute to data heterogeneity, even conflicting data, available in literature and contributed to define the objective of our study.

Starting from these biological supports, a detrimental role of sortilin for diabetic patient has been previously confirmed [20, 31]. Furthermore, sortilin levels correlate with the presence of PAD and PAD severity in a cohort of diabetic patients [21]. Here we found that sortilin levels at baseline are higher in patients with lower ABI. These results are in line with previous data [21] and support the conclusion that an association exists between serum levels of sortilin serum levels and PAD severity in T2DM patient. However, the most relevant result of our research is that in the group of patients with higher sortilin levels at baseline, MACE incidence was more frequent, during the 12 months follow-up, compared with the lower sortilin level group.

The finding is strengthened and confirmed for several reasons. First, the different components of the composite outcome, death, CAD and CVD, showed a similar relationship with baseline serum protein levels. In addition, the ROC curve demonstrated sortilin’s predictive capability of MACE. Finally, multivariate analysis adjusted for traditional cardiovascular risk factors confirmed sortilin’s independent relationship with MACE, death, CAD and CVD. Several possible explanations underlie our data. Sortilin can directly induce a robust inflammatory response [19, 32], because the protein is involved in the interferon-γ, interleukin-6 and toll-like receptor pathways, leading to an important activation of macrophages [19, 32, 33]. In addition to the pro-atherogenic effect, sortilin could induce atherosclerotic plaque instability through inflammatory milieu spread. Plaque formation and subsequent instability could determine the incidence of MACE after LER. Indeed, this suggestion is supported by previous data showing an association between sortilin and CAD, both in diabetic and non-diabetic populations [11, 26].

Sortilin levels, to stratify the initial risk of MACE after LER, are an important new biomarker for clinicians who follow T2DM patients. In fact, up to now, it is impossible to predict the likelihood that patients experience MACE hampering personalized follow-up. Our findings give hope that increasing surveillance after LER in patients with high baseline sortilin levels becomes achievable. Considering also the relationship between sortilin and PCSK9, it is reasonable to use early PCSK9 inhibitors in patients with high sortilin levels.

The secondary objective of this study was to explore the association between the basal levels of sortilin and the development of MALE after LER showing that the group of patients with higher sortilin levels had a higher incidence of MALE during the follow-up. Several T2DM patients with PAD and CLTI developed arterial stenosis recurrence after LER and required frequent endovascular treatments. Numerous studies have been carried out which attempted to identify predictive indicators for MALE after LER. Some encouraging data have been drawn from the prospective assessment of selected inflammatory cytokines [7]. However, definitive biomarkers are not established yet. Sortilin could be included to the inflammatory panel used to stratify cardiovascular risk, since it is involved in the inflammatory processes correlated to atherosclerosis. Considering it as a biomarker of cardiovascular events, the ROC analysis has highlighted that sortilin has a remarkable predictive capacity of MACE and MALE in our population. The AUC resulting from our analysis, in particular for the MACE outcome, is significant and is not prejudiced by the cutoff values, which are usually chosen with conventional approaches of sensitivity and specificity.

Our study has several limitations. First, the patient cohort is relatively small and the results need to be confirmed on a larger number of patients. The small sample size also depends on the restrictive inclusion criteria, which, on the other hand, represent a strength of the study. A further limitation is that few events have occurred in patients with sortilin levels in the lower tertile; therefore, it is not possible to assert with certainty that reduced sortilin levels are protective as higher levels are predictive of cardiovascular events. Furthermore, we have included only statin-free patients, to reduce bias on sortilin levels correlated to different therapies. However, after LER, all patients started lipid-lowering therapy to reach the LDL-C target suggested by international guidelines, which might have influenced circulating sortilin levels during follow-up—a factor not evaluate in our study. Furthermore, considering the extent of the follow-up, it is possible that different lipid-lowering therapies have led to different effects on sortilin levels. However, it is unlikely that they played a direct role on the incidence of MACE, considering that the study population had moderate baseline LDL-C levels. Furthermore, sortilin has been shown to affect the PCSK9 pathway with a mechanism independent of statin therapy. Importantly, our primary objective was to study the relationship between baseline values and MACE development after LER, to identify a possible risk stratification biomarker. In this sense, our results are statistically relevant. A further limitation of our study is that we have not studied the relationship between the initial arterial lesion and the occurrence of subsequent MALE. Therefore, we excluded patients with primary LER failure from the study to decrease bias. Moreover, we did not stratify patients based on MALE type, also given the small number of patients.

It is not clear where sortilin can originate in our patient population. Sortilin derives from pro-sortilin, which is converted into the Golgi apparatus of many cell types. This protein has been found abundantly in the central nervous system, but also in the spinal cord, skeletal muscle, testes, heart, placenta, pancreas, prostate, and small intestine [9]. Previous data suggest that hepatic sortilin is associated with a less dangerous lipid profile [34, 35]; however, investigations on animal models have shown that plasma sortilin could play a harmful role on atherosclerosis development, by non-LDL-C-dependent mechanisms [25]. Nevertheless, there are no convincing data on the source of sortilin in serum. Some reports have shown that increased sortilin levels are associated with depression, increased alcohol intake and BMI [36]. In this scenario, it could be speculated that sortilin may worsen cardiovascular outcomes also by acting on other risk factors. However, we have not studied depression in our patient population, we cannot confirm this hypothesis and further studies are necessary and warranted.

## Conclusion

There are currently no effective biomarkers available to predict the incidence of vascular complications after revascularization of the lower limbs in diabetic patients with CLTI. We still do not know why patients with similar clinical characteristics and those who undergo the same endovascular treatment face completely different complications. In this scenario, biomarkers able to stratify risk and predict the incidence of cardiovascular events are required. In this study, we demonstrated that basal sortilin levels can predict the incidence of MACE and MALE in diabetic patients. The predictive significance of the protein is independent of the classic cardiovascular risk factors. The ability to more effectively predict the onset of complications allows designing a closer and more effective personalized follow-up to avoid potentially fatal vascular events. Although further studies are needed to confirm this data, our findings shed new light on the follow-up management for diabetic patients after LER.

## Supplementary information

**Additional file 1:** **Figure S1.** Graphical representation of the main clinical characteristics of the study populations. **Table S1. **Multivariable logistic regression for Death. **Table S2.** Multivariable logistic regression for CAD. **Table S3.** Multivariable logistic regression for CVD.

## Data Availability

The datasets generated during the current study are available from the corresponding author on reasonable request.

## Abbreviations

ABI: Ankle/brachial index
AUC: Area under the curve
BMI: Body mass index
CAD: Coronary artery disease
CLTI: Chronic limb-threatening ischemia
CVD: Cerebrovascular disease
DAPT: Double antiplatelet therapy
eGFR: Estimated glomerular filtration rate
LDL: C low-density lipoprotein cholesterol
LER: Lower limb revascularization
MACE: Major adverse cardiovascular events
MALE: Major adverse limb events
PAD: Peripheral artery disease
ROC: Receiver operating characteristics
T2DM: Type 2 diabetes mellitus
US: Ultrasound
WIfI: Wound, ischemia, foot infection
