# Supplementary material for: Sortilin levels correlate with major cardiovascular events of diabetic patients with peripheral artery disease following revascularization: a prospective study
Source: Cardiovasc Diabetol. 2020 Sep 25;19:147. doi: 10.1186/s12933-020-01123-3 (PMC7519536; doi:10.1186/s12933-020-01123-3)
Supplement: Supplementary file 1 — Additional file 1: Figure S1. Graphical representation of the main clinical characteristics of the study populations. Table S1. Multivariable logistic regression for Death. Table S2. Multivariable logistic regression for CAD. Table S3. Multivariable logistic regression for CVD. [file 12933_2020_1123_MOESM1_ESM.docx]

**
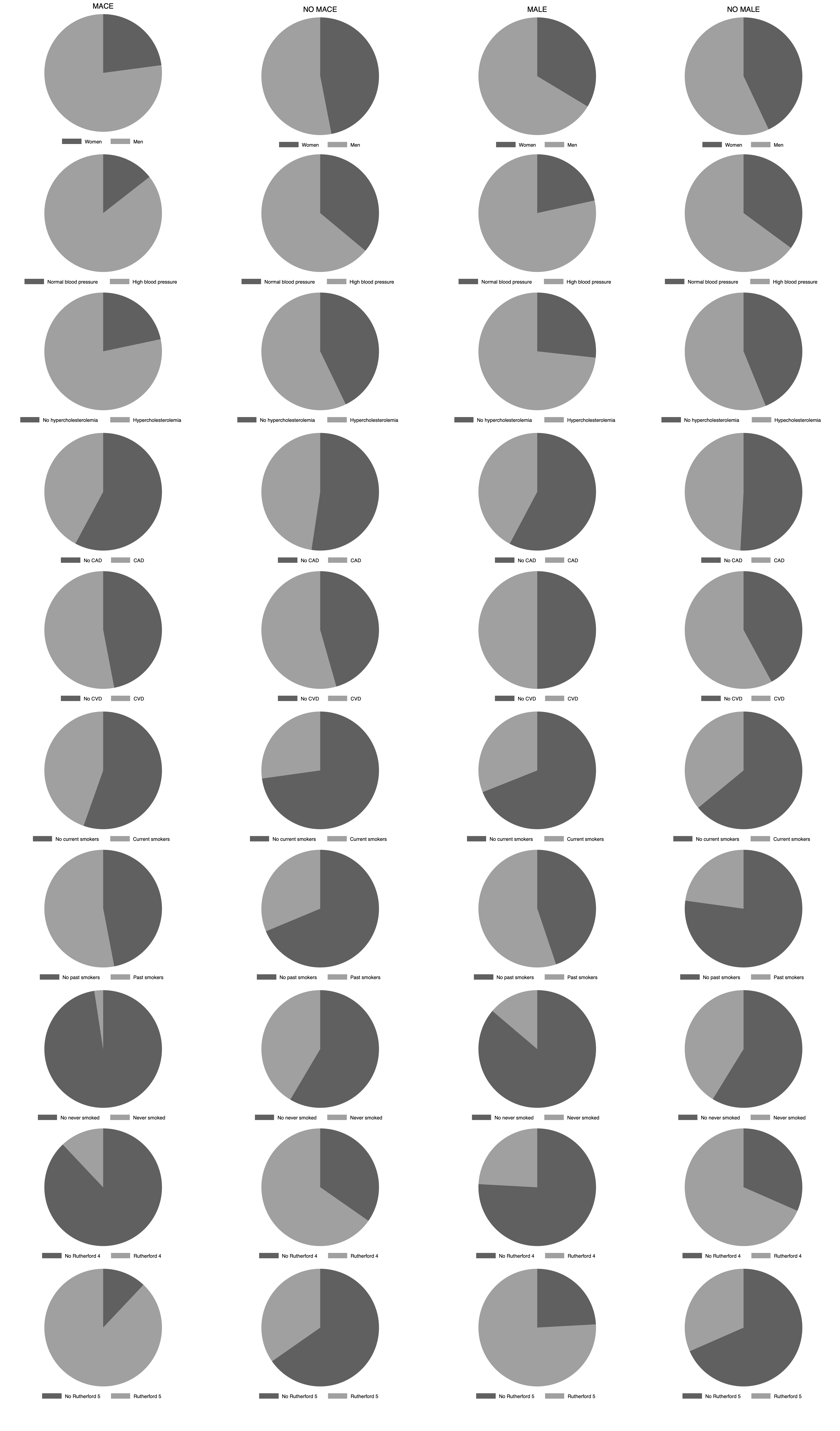
**

**Supplemental Figure 1**

Graphical representation of the main clinical characteristics of the study populations.

**Supplemental Table 1. Multivariable logistic regression for Death**

|  | Coef. | | St.Err. | t-value | | p-value | [95% Conf | | Interval] | | Sig |
| --- | --- | --- | --- | --- | --- | --- | --- | --- | --- | --- | --- |
| Age | 0 | | 0.002 | -0.20 | | 0.839 | -0.004 | | 0.004 | |  |
| Male sex | 0.048 | | 0.038 | 1.25 | | 0.212 | -0.028 | | 0.124 | |  |
| High blood pressure | 0.051 | | 0.042 | 1.22 | | 0.223 | -0.031 | | 0.134 | |  |
| Hypercholesterolemia | 0.009 | | 0.039 | 0.23 | | 0.818 | -0.068 | | 0.087 | |  |
| CAD | -0.013 | | 0.037 | -0.37 | | 0.714 | -0.086 | | 0.059 | |  |
| CVD | 0.038 | | 0.037 | 1.03 | | 0.304 | -0.034 | | 0.11 | |  |
| Current smokers | 0.087 | | 0.054 | 1.61 | | 0.109 | -0.02 | | 0.194 | |  |
| Past smokers | -0.11 | | 0.055 | -1.99 | | 0.047 | -0.218 | | -0.001 | | ** |
| Never smoked | 0 | | . | . | | . | . | | . | |  |
| LDL-C | 0.004 | | 0.001 | 3.58 | | 0 | 0.002 | | 0.006 | | *** |
| FPG | 0.003 | | 0.001 | 1.91 | | 0.057 | 0 | | 0.005 | | * |
| HbA1C | 0.018 | | 0.009 | 1.97 | | 0.05 | 0 | | 0.037 | | * |
| Sortilin | 0.091 | | 0.039 | 2.32 | | 0.021 | 0.014 | | 0.169 | | ** |
| Constant | -1.047 | | 0.263 | -3.99 | | 0 | -1.565 | | -0.529 | | *** |
|  | | | | | | | | | | | |
| Mean dependent var | | 0.104 | | | SD dependent var | | | 0.306 | |  |  |
| R-squared | | 0.270 | | | Number of obs | | | 230.000 | |  |  |
| F-test | | 6.673 | | | Prob > F | | | 0.000 | |  |  |
| Akaike crit. (AIC) | | 61.314 | | | Bayesian crit. (BIC) | | | 106.009 | |  |  |
| **** p<.01, ** p<.05, * p<.1* | | | | | | | | | | | |
|  | | | | | | | | | | | |

**Supplemental Table 2. Multivariable logistic regression for CAD**

|  | Coef. | | St.Err. | t-value | | p-value | [95% Conf | | Interval] | | Sig |
| --- | --- | --- | --- | --- | --- | --- | --- | --- | --- | --- | --- |
| Age | 0 | | 0.002 | -0.17 | | 0.869 | -0.005 | | 0.004 | |  |
| Male sex | 0.102 | | 0.046 | 2.21 | | 0.028 | 0.011 | | 0.193 | | ** |
| High blood pressure | 0.018 | | 0.05 | 0.35 | | 0.723 | -0.081 | | 0.117 | |  |
| Hypercholesterolemia | 0.003 | | 0.047 | 0.07 | | 0.947 | -0.09 | | 0.096 | |  |
| CAD | -0.062 | | 0.044 | -1.40 | | 0.162 | -0.15 | | 0.025 | |  |
| CVD | 0.001 | | 0.044 | 0.02 | | 0.982 | -0.086 | | 0.088 | |  |
| Current smokers | -0.041 | | 0.065 | -0.63 | | 0.531 | -0.17 | | 0.088 | |  |
| Past smokers | -0.067 | | 0.066 | -1.01 | | 0.311 | -0.197 | | 0.063 | |  |
| Never smoked | 0 | | . | . | | . | . | | . | |  |
| LDL-C | 0.006 | | 0.001 | 4.80 | | 0 | 0.004 | | 0.009 | | *** |
| FPG | 0.002 | | 0.002 | 1.03 | | 0.305 | -0.002 | | 0.005 | |  |
| HbA1C | -0.001 | | 0.011 | -0.10 | | 0.922 | -0.023 | | 0.021 | |  |
| Sortilin | 0.33 | | 0.047 | 6.95 | | 0 | 0.236 | | 0.423 | | *** |
| Constant | -1.259 | | 0.316 | -3.99 | | 0 | -1.882 | | -0.637 | | *** |
|  | | | | | | | | | | | |
| Mean dependent var | | 0.261 | | | SD dependent var | | | 0.440 | |  |  |
| R-squared | | 0.488 | | | Number of obs | | | 230.000 | |  |  |
| F-test | | 17.230 | | | Prob > F | | | 0.000 | |  |  |
| Akaike crit. (AIC) | | 146.195 | | | Bayesian crit. (BIC) | | | 190.890 | |  |  |
| **** p<.01, ** p<.05, * p<.1* | | | | | | | | | | | |
|  | | | | | | | | | | | |

**Supplemental Table 3. Multivariable logistic regression for CVD**

|  | Coef. | | St.Err. | t-value | | p-value | [95% Conf | | Interval] | | Sig |
| --- | --- | --- | --- | --- | --- | --- | --- | --- | --- | --- | --- |
| Age | -0.002 | | 0.003 | -0.91 | | 0.366 | -0.008 | | 0.003 | |  |
| Male sex | 0.068 | | 0.052 | 1.31 | | 0.192 | -0.034 | | 0.17 | |  |
| High blood pressure | -0.008 | | 0.056 | -0.15 | | 0.881 | -0.12 | | 0.103 | |  |
| Hypercholesterolemia | -0.041 | | 0.053 | -0.78 | | 0.435 | -0.146 | | 0.063 | |  |
| CAD | 0.002 | | 0.05 | 0.04 | | 0.966 | -0.096 | | 0.1 | |  |
| CVD | 0.048 | | 0.049 | 0.97 | | 0.335 | -0.05 | | 0.145 | |  |
| Current smokers | 0.064 | | 0.073 | 0.88 | | 0.379 | -0.08 | | 0.209 | |  |
| Past smokers | -0.076 | | 0.074 | -1.03 | | 0.306 | -0.222 | | 0.07 | |  |
| Never smoked | 0 | | . | . | | . | . | | . | |  |
| LDL-C | 0.005 | | 0.001 | 3.28 | | 0.001 | 0.002 | | 0.008 | | *** |
| FPG | 0.003 | | 0.002 | 1.42 | | 0.158 | -0.001 | | 0.006 | |  |
| HbA1C | -0.001 | | 0.013 | -0.06 | | 0.95 | -0.026 | | 0.024 | |  |
| Sortilin | 0.231 | | 0.053 | 4.35 | | 0 | 0.126 | | 0.336 | | *** |
| Constant | -0.932 | | 0.354 | -2.63 | | 0.009 | -1.629 | | -0.235 | | *** |
|  | | | | | | | | | | | |
| Mean dependent var | | 0.226 | | | SD dependent var | | | 0.419 | |  |  |
| R-squared | | 0.292 | | | Number of obs | | | 230.000 | |  |  |
| F-test | | 7.450 | | | Prob > F | | | 0.000 | |  |  |
| Akaike crit. (AIC) | | 198.442 | | | Bayesian crit. (BIC) | | | 243.137 | |  |  |
| **** p<.01, ** p<.05, * p<.1* | | | | | | | | | | | |
|  | | | | | | | | | | | |
